# New missions of a hospital pharmaceutical technology unit during the COVID-19 pandemic

**DOI:** 10.1186/s40545-020-00283-7

**Published:** 2021-01-06

**Authors:** Alexandra Garnier, Ludivine Falaschi, Pascal Bonnabry, Lucie Bouchoud

**Affiliations:** 1 Geneva University Hospitals, Street Gabrielle-Perret-Gentil 4, 1205 Geneva, Switzerland; 2 Institute of Pharmaceutical Sciences of Western Switzerland, School of Pharmaceutical Sciences, University of Geneva, Street Michel-Servet 1, 1205 Geneva, Switzerland

**Keywords:** COVID-19, Pharmaceutical technology, Hydroalcoholic solution, Human resources

## Abstract

The pharmaceutical technology unit of the Geneva University Hospitals played a significant role in the fight against COVID-19 through four different missions: (1) providing enough hydroalcoholic solution at the peak of the pandemic; (2) facing supply chain management issues; (3) adapting the workload to the crisis and, above all, (4) managing the human resources necessary to handle these activities.

## Introduction

On March 16th 2020, the Federal Council declared Switzerland to be in an “extraordinary situation”. The Geneva University Hospitals (HUG) is one of the five university hospitals of Switzerland and the largest one in the country with 2000 beds and 11,000 employees. In the same way as many hospital pharmacists fought the COVID-19 pandemic [1], HUG’s pharmaceutical technology unit (PTU) took new responsibilities.

## Mission 1: providing hydroalcoholic solution

At the peak of the pandemic, the HUG’s consumption of hydroalcoholic solution was multiplied by three. The external supplier was overwhelmed and a shortage in hydroalcoholic solution was threatening. Therefore, the PTU started a production chain on March 6th, 1 month before the most critical time of the pandemic. Within 3 weeks, 1120 L had been produced as per the OMS formula [2]. In April, Firmenich, a Swiss fragrance and flavor company, decided to use its own chain to produce and send cans of hydroalcoholic solution to the pharmacy, to be conditioned in 100-mL and 500-mL bottles and distributed. On August 11th, 10,000 L of hydroalcoholic solution had been filled in bottles which were closed, labeled, stored and sent to hospital care units. This unusual situation should go back to normal in November, when the supplier is able to furnish enough hydroalcoholic solution again. Considering that it was impossible to obtain anywhere such quantities of hydroalcoholic solution and that 6 months were necessary for the supplier to adapt his production, these reactivity and anticipation were primordial and proved to be incredibly useful.

Part of this mission was the recycling of used bottles which were washed and filled again. Collection points were efficiently organized and the entire hospital participated in this collective effort.

## Mission 2: facing supply chain management issues

In HUG, the demand in reanimation drugs increased by ten times during the pandemic, although the world was facing shortages related to the lack of active pharmaceutical ingredients from China, the first country to be hit by the virus. To face these supply chain management issues, the pharmacy switched to unusual suppliers and received drugs from various non-French-speaking countries. In a very short time, the PTU handled the relabeling of 21,000 ampules of atracurium from Portugal, 20,000 ampules of propofol for Germany, 5000 vials of Esmeron from South Korea and 220 vials of remdesivir initially used for a clinical trial.

## Mission 3: adapting usual missions

Several clinical activities in the hospital were reduced or stopped to take over COVID+ patients, leading to: (1) reevaluating the need of some planned serial productions; (2) suspending clinical trials; (3) postponing some chemotherapy treatments and (4) stopping autologous serum production. A global shortage in sampling tests lead to a collaboration with the virology laboratory to develop a DMEM culture medium used for the PCR screening of COVID-19 cases. Therefore, this saved time was reinvested to fill 30′000 sterile tubes of this reactant, representing more than 130 h of work on 31 days.

## Mission 4: preserving human resources

Guaranteeing the continuity of human resources by identifying key functions and vulnerable employees was a challenge. Every holiday until April 30th were canceled on March 13th. Not to exhaust the team, recovery days were maintained at the PTU. Usually, the unit works with a minimum of 7 technicians, 2 assistant-technicians and 2 pharmacists. For a worst-case scenario, the minimum was decided to be 4 technicians, 1 assistant-technician and 1 pharmacist during a few days versus 5 technicians, 2 assistant-technicians and 1 pharmacist if the crisis lasted for several weeks.

Other options were considered: (1) increasing some employees’ work rate; (2) collaborating with community pharmacies or the hospital of Vaud canton; (3) having technicians back from other units and (4) calling back some retirees. The first option was applied and one part-time collaborator worked on a 100% basis during one month. Furthermore, 11 volunteers joined the PTU, furnishing the equivalent of 90 days of work, essentially used for the filling of hydroalcoholic solution.

Pharmacists also started to work remotely with several missions: (1) double control of volumes with pictures; (2) liberation of pharmaceutical preparations; (3) creation and validation of protocols; (4) phone calls to suppliers and (5) invoice of cytotoxic preparations to care units.

## Conclusion

The HUG’s PTU has faced many challenges during the COVID-19 pandemic. From historical perspective, it is an unprecedented situation that can be now considered as a great opportunity to apply in the future the keywords adaptation, resilience and rigor.

## Data Availability

The datasets used in this paper are available from the corresponding author.
